# Autoantibodies as Potential Biomarkers in Breast Cancer

**DOI:** 10.3390/bios8030067

**Published:** 2018-07-13

**Authors:** Jingyi Qiu, Bailey Keyser, Zuan-Tao Lin, Tianfu Wu

**Affiliations:** Department of Biomedical Engineering, University of Houston, 3517 Cullen BLVD, SERC 2008, Houston, TX 77204, USA; yva1ne@hotmail.com (J.Q.); bbkeyser@central.uh.edu (B.K.); zuantaolin@gmail.com (Z.-T.L.)

**Keywords:** autoantibody, breast cancer, early diagnosis, immunotherapy

## Abstract

Breast cancer is a major cause of mortality in women; however, technologies for early stage screening and diagnosis (e.g., mammography and other imaging technologies) are not optimal for the accurate detection of cancer. This creates demand for a more effective diagnostic means to replace or be complementary to existing technologies for early discovery of breast cancer. Cancer neoantigens could reflect tumorigenesis, but they are hardly detectable at the early stage. Autoantibodies, however, are biologically amplified and hence may be measurable early on, making them promising biomarkers to discriminate breast cancer from healthy tissue accurately. In this review, we summarized the recent findings of breast cancer specific antigens and autoantibodies, which may be useful in early detection, disease stratification, and monitoring of treatment responses of breast cancer.

## 1. Introduction

Breast cancer is the prevailing cancer among women in developing and developed countries [1]. As such, screening and early diagnosis with respect to risk stratification are critical for prevention and early intervention of the disease, leading to better therapeutic outcomes [2,3]. Breast cancer itself is genetically heterogeneous and expresses a variety of aberrant proteins that, until recently, were un-utilizable. Of the current commercially available detection methods, mammography is the only screening technology to improve mortality; however, there are clear limitations to the technology [4]. Initially, mammography lacks sensitivity, rendering the technology less beneficial in younger women (ages 40–49 years) [5,6] and, most importantly, ill-suited for detection of node-negative early-stage (T1N0) primary breast cancer (PBC) and ductal carcinoma in situ (DCIS) [7,8]. Additional studies have indicated that mammography can lead to false positive results, despite the poor sensitivity, especially for women who started screening at young ages due to the larger number of mammograms and subsequently higher recall rate [9]. As a consequence, overdiagnosis by mammograms occurs within 1–10% of patients [10]. Additionally, magnetic resonance imaging (MRI) and breast ultrasound, which also contribute to false positives, are used as supplementary diagnosis methods for patients with invasive breast cancer after an initial screening mammography [11]. Recently, however, with the development of next-generation sequencing (NGS) and complementary advanced sequencing technologies, these aforementioned mutated cancer gene sequences are detectable in blood samples from patients and through techniques such as liquid biopsy [12], circulating tumor DNA (ctDNA) [13,14], and cell-free DNA (cfDNA) [15]. However, given the low concentrations of these ctDNAs, amplification steps are usually necessary, complicating and prolonging the detection protocols.

Autoantibodies (AABs) present an alternative to the above with the advantage of minimalist hardware, short time-frames (30 min), and an abundance of targets [16]. These proteins are a selection of antibodies whose associated antigens are produced by the organism’s own cells; the antigens themselves are expressed at low concentrations in healthy cells and overexpressed or aberrantly expressed in cancerous cells. This erroneous expression of antigens is detected by the immune system through methods such as toll-like receptors (TLRs) for the innate response, with the potential to both exacerbate and reverse tumor growth, and the class 2 major histocompatibility complex (MHC) by phosphorylation and or glycosylation of the “normally” expressed antigen, preventing recognition [17,18]. MUC1, for example, an integral membrane protein of the breast, was detected to be overexpressed in 90% of adenocarcinomas and was further linked to tumor aggression [17,19]. Unfortunately, the immune surveillance of breast cancers has been found to be compromised as the IRF7 pathway inside of breast cancer cells is suppressed. This is mitigated by the multicomponent nature of said surveillance system and the fact that the same pathway also governs the metastasis of the cancer alongside the TLRs, allowing for more accurate prognostics [18,20].

Ultimately, the ideal test would accurately identify breast cancers that require intervention, preferably at an early stage, minimizing the amount of surgical and pharmacological treatment, while avoiding overdiagnosis and subsequent overtreatment [21]. Therefore, efficient, low-cost, and highly sensitive technology is needed, particularly for women with early-stage breast cancer.

## 2. Autoantibodies in Breast Cancer

Current blood-based detection assays are ill-suited for screening, classification, and impacting treatment decisions [22]. This is a consequence of the early breast cancer stages being characterized by minimal disease burden—less than 1 × 10^6^ tumor cells—and low or undetectable serum levels of protein biomarkers, making the monitoring of traditional biomarkers challenging.

**Figure 1:**
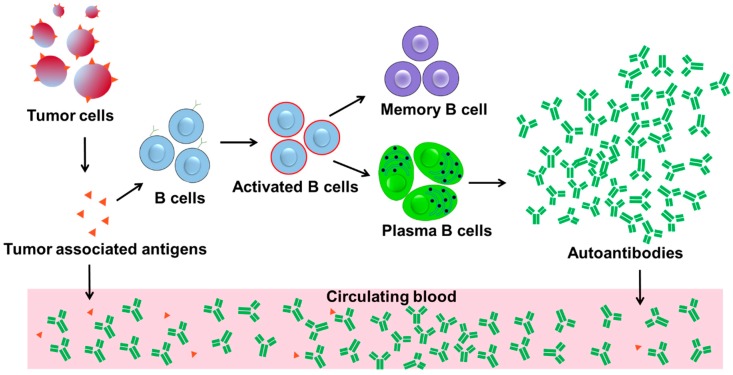
Schematic diagram of autoantibody production and amplification by cancer antigen stimulation. B cells produce many copies of autoantibodies during immune responses, which amplify the cancer antigen “signal”, becoming detectable during early-stage breast cancer. In comparison, the concentration of antigens is too low to be detected in the same timeframe.

B cells, however, offer a solution to the above problem by class switching to produce high affinity matured autoantibodies, massively abundant high affinity biomarkers, in response to the presence of antigens, which occurs during the early stages of cancers as shown in Figure 1. An individual B cell can produce 5000–20,000 antibodies/min, as well as undergo mitosis every 3 days, further maintaining or enhancing autoantibody production [23,24,25]. The specificity of B cell autoantibody responses to tumor antigens and effective amplification of “tumor signal” can fulfill the desirable features of a biomarker, namely specificity and sensitivity (Figure 1). In fact, specific immune escape events may herald the transition from in situ to invasive breast cancer, which suggest that autoantibody reaction-based assays can be effective for early detection [26]. In particular, the IgG κ chain has been indicated as a prognostic biomarker in breast cancer, with applications in predicting disease response and neoadjuvant chemotherapy efficacy [27,28]. Interestingly, clinical trials have demonstrated higher response rates to immune checkpoint blockade in triple-negative breast cancer (TNBC) tumors when compared with estrogen-receptor positive (ER+) tumors, allowing for potential discrimination [29,30,31].

Furthermore, gene mutations lead to successive generations of neoantigens, while malignant transformation and the associated apoptosis will release excessive antigens. These antigens may trigger the immune system to produce high titers of autoantibodies or tumor-associated autoantibodies (TAABs). These TAABs can be promising biomarkers for early diagnosis of breast cancer based on concentration, which may precede clinical confirmation of cancer by months to years, as the detection of autoantibodies can be performed earlier than the originating tumor-associated antigens (TAAs) assays [2,32,33]. Moreover, there are a plethora of TAABs, which are highly stable in serum and whole-blood compared to other polypeptides [34]. These autoantibodies against TAAs have been verified in breast cancer, as summarized in Table 1. As such, using autoantibody biomarkers to achieve early diagnosis of breast cancer is promising and can reflect clinical responses to immunotherapy, as has been shown in several malignancies [35,36,37]. Therefore, immunoreactive autoantibodies (IR-Abs) of TAAs provide an in vivo amplification of early cancer signals and allow for earlier detection. Furthermore, there is evidence for a specific humoral response against a number of intracellular and surface tumoral antigens related to breast cancer [38,39,40,41,42,43,44,45,46,47,48,49,50,51]. However, these results did not lead to clinically useful biomarkers for early diagnostics of breast cancer due to relatively poor sensitivity and specificity.

An example of a useful autoantibody is heat-shock protein 60 (HSP60), positive in 31% of patients during early-stage breast cancer and 32.6% of patients with DCIS, with a miniscule 4.3% presentation in healthy controls [52,53], which is consistent with Hamrita et al.’s detection of HSP60 in 19 out of 40 invasive breast cancer patients (47.5%) and only 2 out of 42 healthy controls (4.7%) [54]; together, these studies strongly indicate that HSP60 may be a potential TAA for the diagnosis of noninvasive and invasive ductal carcinoma. Additionally, several other autoantigens may be involved in the pathways of breast cancer tumorigenesis, such as those found in the mammalian target of the rapamycin (mTOR) phosphorylation pathway: ribosomal protein S6, eukaryotic elongation factor 2, eukaryotic elongation factor 2 kinase, heat shock protein 90 (HSP90), and the DNA damage/repair pathways, such as Ku protein, topoisomerase I, and the 32-kDa subunit of replication protein A [52,55]. Other proteins not involved in the above pathways have also been found to present in altered expression but have not been validated. A few of these proteins are as follows: keratins, actins, histones, serine/arginine splicing factors, and Ubiquitin [56]. Ultimately, a proteomic autoantibody screening method can open new avenues in uncovering molecular mechanisms of tumorigenesis for breast cancer of varying types. As detailed in a recent systematic review and meta–analysis by Xia and colleagues, autoantibodies against p53, MUC1, HER2, and cyclin B1 are the top 4 among all breast cancer-associated autoantibodies in terms of the frequency of studies. There are considerable variations in terms of sensitivity and specificity of these autoantibodies as potential biomarkers of breast cancer in disease diagnosis [57], which may be due to different study sites, different assay technologies and platforms, different experimental procedures and protocols, and different patient populations. Therefore, it should be pointed out that it is too early to rank the diagnostic values or predictive values of these autoantibodies for clinical use. Standardized assay protocols may be needed and may be helpful in establishing highly accurate and robust autoantibodies as biomarkers of breast cancer. Although the exact function of these autoantibodies is not clear, we could speculate that these autoantibodies may be generated by B cells to neutralize cancer promoting proteins/neo-antigens, in order to suppress cancer growth.

## 3. Autoantibody Detection in Breast Cancer

Several technologies such as immunosensors, enzyme-linked immunosorbent assay (ELISA), antigen arrays, and bead arrays have been applied to the detection of autoantibodies in breast cancer patients. Immunosensors have been demonstrated to detect autoantibodies in autoimmune disease and have the potential for similar detection of autoantibodies in breast cancer [58]. ELISA, the more traditional immunological assay, has been successfully used to measure the concentration of autoantibodies in breast cancer patients (Table 1). Antigen arrays, an efficient high-throughput technology, can also be used for the same task. As such, technologies like ELISA and antigen arrays can be applied as complements in breast cancer studies. As an example, antigen arrays can be used to discover potential autoantibodies in breast cancer against hundreds and thousands of antigens. After the establishment of an antigen–autoantibody pair, ELISA can be applied for a large cohort of patients and controls so that statistical analysis can be applied to extract meaningful data.

An example of this is HER-2/neu, a breast cancer specific antigen, which Disis et al. identified using ELISA and western blot techniques. Then they discovered autoantibodies against HER-2/neu at the early-stage of breast cancer. These HER-2/neu antibodies in breast cancer patients were correlated with HER-2/neu protein expression and HER-2/neu-positive cancer [59]. Additionally, Mudenda and colleagues reported that autoantibodies to p53 were found in breast cancer patients in all stages of disease progression. Interestingly, in a longitudinal cohort of breast cancer patients, p53 autoantibody levels positively correlated with histology grades and p53 expression in cancer tissues [60].

However, there is no single autoantibody found, which has been used as a clinical biomarker—a consequence of the heterogeneous nature of breast cancer. The proteins in said cancer are aberrantly expressed either post-translationally modified or irregularly regulated in the same type of cancer [52,78]. It has become obvious that a single autoantibody biomarker is not sufficient to provide information about tumor progression [79]. Therefore, a combination of selected autoantibodies arranged as a biomarker panel may be more attractive. Although such an autoantibody panel is not yet available, a bead array panel of 35 tumor-associated antigens was constructed by Kim et al. [43], which possessed a high accuracy of 91.8% by random forest analysis, 91.5% by support vector machine analysis, and 87.6% by linear discriminant analysis in distinguishing breast cancer patients from healthy controls [43].

It should be noted that the cancer antigen 15-3 (CA15-3) and the carcinoembryonic antigen (CEA) have been approved by Food and Drug Administration (FDA) as tumor markers for breast cancer. Additionally, CA125 and malignant tumor-specific growth factor (TSGF) have been suggested as tumor-associated markers [80,81,82]. The investigation of autoantibodies against these cancer antigens can provide valuable information in uncovering disease mechanisms, identifying novel drug targets and establishing diagnostic biomarkers for breast cancer.

## 4. Technologies for Autoantibody Discovery and Detection in Breast Cancer

During the past decade, several high-throughput technologies have been developed and utilized in the discovery and detection of autoantibodies. Technologies, such as serological analysis of tumor antigens by recombinant cDNA expression cloning (SEREX) [52,83,84], phage display [85,86,87], serological proteome analysis (SERPA) [54,88,89], multiple affinity protein profiling (MAPPing) [90], protein microarrays [91,92,93,94,95,96], and nanoplasmonic sensors [16] have been applied in the study of autoantibody biomarkers of breast cancer [78].

### 4.1. Serological Analysis of Tumor Antigens by Recombinant cDNA Expression Cloning (SEREX)

In this technology, TAABs are identified by screening patient sera against a cDNA expression library obtained from the autologous tumor tissues [97]. Over 2000 autoantigens are documented in an online database, the Cancer Immunome Database (CID) [98,99,100]. SEREX facilitated the identification of TAAs as possible cancer biomarkers in different types of cancer, such as lung, liver, breast, prostate, ovarian, renal, head, neck, esophageal cancers, leukemia, and melanoma [52,101]. The panel of SEREX-defined immunogenic tumor antigens include Cancer/Testis Antigens (CTAs, e.g., NY-ESO-1, SSX2, MAGE), mutational antigens (e.g., p53), differentiation antigens (e.g., tyrosinase, SOX2, ZIC2), and embryonic proteins [52]. The disadvantage of SEREX lies in that it is time-consuming, labor-intensive and difficult to automate for high-throughput assay. In addition, autoantibodies against post-translational modifications of antigens cannot be detected by SEREX. 

**Figure 2:**
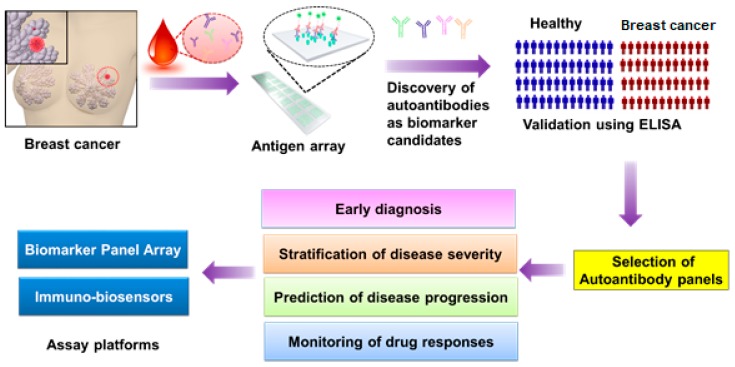
Flowchart of autoantibody biomarker discovery and detection in breast cancer using antigen arrays and ELISA. First, a drop of blood from breast cancer (BC) patients is subjected to an antigen array for a high-throughput screening of autoantibodies that specifically bind to breast cancer antigens on an array. Second, promising autoantibody candidates are selected from the array screening and validated in a large cohort of patients using ELISA, which can be used for early diagnosis, disease stratification, prediction of disease progression, or monitoring of drug responses. Finally, according to the function of each autoantibody biomarker or biomarker panel, biosensors or autoantigen-panel chips could be designed and fabricated for clinical use in breast cancer.

There is no doubt that individual assays, such as ELISA, immunosensors [58], or multiplexing bead-based autoantibody assays are important tools for the detection and validation of the level of specific autoantibodies as biomarkers, especially in large cohorts of patients for diagnosis and disease monitoring of breast cancer, as illustrated in Figure 2.

### 4.2. Serological Proteome Analysis (SERPA)

SERPA is a proteomic approach which combines two-dimensional (2D) electrophoresis, western blotting, and mass spectrometry (MS) [52,88]. Briefly, proteins from tissue or cells are separated by isoelectric and SDS-PAGE gel electrophoresis and transferred onto membranes, followed by probing with sera from patients with cancer. Subsequently, the differentially expressed immunoreactive cancer antigens are excised from the gel and identified by MS. Klade et al. developed SERPA, and identified carbonic anhydrase I (CAI) and smooth muscle protein 22 (SM22) in kidney cancer tissues [102]. Kellner et al. showed that several members of the cytoskeletal family (such as cytokeratin 8, stathmin, and vimentin) are potential TAABs that can distinguish between renal cell carcinoma subtypes and from the normal renal epithelium tissue [103]. Furthermore, when coupled with western blotting for serological screening, 2D gel could be used to detect TAABs that undergo post-translational modifications via MS analysis.

SERPA has been applied in the discovery of autoantibodies in various cancer types, including neuroblastoma, lung cancer, breast cancer, renal cell carcinoma, hepatocellular carcinoma (HCC), and ovarian cancer [104,105,106,107,108]. This technique eases the detection of novel autoantibodies and associated autoantigens as early indicators of tumorigenesis. Several autoantibodies, such as hnRNPK, Mn-SOD, HSP60, and F1-ATPase, were identified in breast cancer using SERPA [54].

### 4.3. Multiple Affinity Protein Profiling (MAPPing)

MAPPing comprises 2D immunoaffinity chromatography followed by the identification of TAABs by tandem mass spectrometry (2D-LC-MS/MS) [90,109]. In immunoaffinity chromatography, TAAs from cancerous tissues bind to IgG from healthy controls. The unbound fraction of the lysate is then subjected to the 2D immunoaffinity column that contains IgG from cancer patients. TAAs that bind are likely to be cancer-specific and are identified by tandem MS/MS [109].

### 4.4. Protein Microarray

A novel high-density custom protein microarray, nucleic acid protein programmable array (NAPPA), is fabricated by printing full-length cDNAs encoding the target proteins, and the target proteins are then transcribed and translated by a cell-free system. Tumor antigens from nearly 5000 breast cancer patients at early stages were detected using NAPPA, and 28 antigens were found to be highly responsive to their relevant autoantibodies: ATP6AP1, PDCD6IP, DBT, CSNK1E, FRS3, RAC3, HOXD1, SF3A1, CTBP1, C15orf48, MYOZ2, EIF3E, BAT4, ATF3, BMX, RAB5A, UBAP1, SOX2, GPR157, BDNF, ZMYM6, SLC33A1, TRIM32, ALG10, TFCP2, SERPINH1, SELL, and ZNF510 [91]. Ola Blixt et al. synthesized MUC1 glycopeptides and used a novel microarray to test a large cohort of breast cancer patients and healthy controls. It was found that early-stage breast cancer has more frequent and higher levels of autoantibodies to glycosylated MUC1 compared to healthy controls, which indicates that autoantibodies may reflect disease progression [61].

### 4.5. Nanoplasmonic Biosensor

The nanoplasmonic biosensor is an etched glass substrate that utilizes the surface plasmons of gold in combination with bound TAAs. The system measures the reflective index of the material at the baseline and as it changes due to local surface bioactivity after the introduction of untreated plasma or sera. The sensitivity of the system is increased through the use of a waveguided light source with limits of detection for GTF2b and EDIL3 antibodies of approximately 10 and 5 ng/mL, respectively. The total time to run the assay is 30 min, and it has the ability to be reused for over 100 cycles [16].

## 5. Look into the Future

So far, most studies on autoantibodies in breast cancer have focused on their diagnostic values. It should be pointed out that autoantibodies, as important indicators of the function of the immune system, will play more important roles in monitoring drug responses, especially immunotherapy. In the past decade, immunotherapy has been successfully applied in metastatic melanoma with strong clinical responses in malignancies, such as lung, kidney, bladder cancers, and non-metastatic melanoma [110]. Current immunotherapy techniques can be divided into two categories: passive and active. Passive immunotherapy has been successfully applied in clinics, where treatments such as Trastuzumab, known as Herceptin, are applied. Trastuzumab is a monoclonal antibody targeting the extracellular domain of the HER2 protein, and it is the mainstay of passive immunotherapy in HER2-positive breast cancer [111]. The antibody can selectively bind to HER2 receptors to prevent breast cancer cells from proliferating, achieving a therapeutic goal. In 2005, Piccart-Gebhart and colleagues found that one-year treatment with Trastuzumab after chemotherapy significantly improved survival rate for HER2-positive breast cancer [112,113]. Active immunotherapy often refers to cancer vaccines. For example, E75, a human leukocyte antigen (HLA)-A2/A3-restricted immunogenic peptide derived from the HER2 protein, has been used as a vaccine to prevent disease recurrence in high-risk breast cancer patients [114]. Recently, an in situ vaccination was used to trigger a T cell immune response to attack cancer cells (e.g., the combination of a Toll-like receptor 9 (TLR) ligand and an anti-OX40 antibody can successfully cure various types of cancer) [115]. This approach was successfully applied in mouse models; however, more time is required for human trials. More recently, immune checkpoint inhibitors have emerged with promise as a cancer treatment [116,117,118,119]. For example, PD1 has been used to block checkpoint inhibitors preventing the proliferation of tumors and showing encouraging anticancer therapeutic effects [116,117,118,119]. Studies on mutational load, immune profile, and response to immune checkpoint inhibition in a BRCA1-deficient tumor models have provided a rationale for clinical studies of combined immune checkpoint blockade in BRCA1-associated TNBC [120].

Tumor-infiltrating lymphocytes (TIL) [121,122], such as cytotoxic T cells, may predict better patient outcomes and responses to drugs (such as checkpoint blockade therapies); whereas, an increased number of regulatory T cells (Treg: CD4^+^FoxP3^+^CD25^high^) or myeloid-derived suppressor cells (MDSC) may correlate with lower survival rates in cancer and with lower clinical response rates to anti-CTLA-4 antibodies [123,124,125,126]. Also, CD20+ B cells among TIL correlated with favorable prognosis in ovarian cancer [127]. These findings suggest that the specific immune status of patients may be indicative of the capability to respond to and suppress tumor progression. However, immune cell-based detection is largely dependent on flow cytometry, a tedious and complicated operation, especially when multiple staining procedures are needed for various cell subsets and activation states.

As an alternative approach, autoantibody levels in the serum of breast cancer patients could potentially be used to monitor treatment responses during immunotherapy. Combination treatment, such as radiation plus chemotherapy or radiation plus hormonal therapy, resulted in a significant decrease of autoantibodies [62], which indicates that immunotherapy may be beneficial to these patients. Future direction can be focused on the real-time monitoring of tumor-associated autoantibody levels, which may aid immunotherapy. 

In conclusion, as the immune system is an indispensable player during tumorigenesis and cancer development, autoantibodies, particularly cancer antigen-specific autoantibodies, may be used as early biomarkers for cancer detection and prevention. More importantly, the detection of these autoantibodies may be indicative of novel treatment strategies (e.g., development of monoclonal antibodies against the same cancer antigen to cure the disease). Also, the development of novel assays for the detection of cancer-specific autoantibodies, such as autoantibody panel array and ultrasensitive immunobiosensors, may provide a powerful complementary strategy to mammography in the screening of suspicious breast cancer patients.

## Figures and Tables

**Table 1:** Tumor-associated autoantibodies in breast cancer.

Autoantibodies/Antigens	Detection Method	Patient Cohort (N)	Reference
ANGPTL4, DKK1, GAL1, MUC1, GFRA1, GRN, and LRRC15	ELISA	Breast cancer (200), controls (200)	[40]
CTAG1B, CTAG2, TP53, RNF216, PPHLN1, PIP4K2C, ZBTB16, TAS2R8, WBP2NL, DOK2, PSRC1, MN1 and TRIM21	Protein array	Basal-like breast cancer (BLBC, 45), controls (45)	[41]
HSPB1, HSPD1, HSP70, HSP90, HSPA5, HSP90B1 and HSP110	Protein microarray	Breast cancer (50), controls (26)	[46]
HER-2/neu	ELISA	Breast cancer (107), healthy controls (200)	[59]
p53	ELISA	Breast cancer (182); Healthy controls (76)	[60]
MUC1	ELISA, Peptide array	Breast cancer (395); Healthy controls (99)	[61]
A1AT, ANGPTL4, CAPC, CST2, DKK1, GFRA1, GRN, LGALS3, LRP10 and GRP78	Luminex multiplex bead assay	Breast cancer, longitudinal (200)	[62]
alpha 2-HS glycoprotein	ELISA	Breast cancer (81), Healthy controls (73)	[63]
HER-2, p53, CEA, Cyclin B1	ELISA, protein array	Breast cancer: controls Training set: 98: 98 Validation Set: 20:20; 33:45	[64]
p53, c-myc, HER-2, NY-ESO-1, BRCA1, BRCA2 and MUC1	ELISA	Primary breast cancer (97), ductal carcinoma in situ (40), normal (94)	[49]
PPIA, PRDX2, and FKBP52	ELISA	Primary breast cancer (60), carcinoma in situ (82), controls (93)	[53]
HSP60	ELISA	Ductal carcinoma in situ (DCIS) (49), early stage breast cancer (58), other cancers (20), healthy controls (93)	[53]
IMP1, p62, Koc, p53, c-myc, surviving, p16, cyclin B1, cyclin D1, and CDK2	Mini-array, ELISA	Breast cancer (41), controls (82)	[65]
CA15-3, LGALS3, PHB2, MUC1, and GK2	Protein array	Breast cancer (100), controls (50)	[66]
alpha-enolase (ENO1)	ELISA	Breast cancer (178), controls (99)	[67]
SOX2	ELISA	Breast cancer (282), benign disease (78), healthy (194)	[68]
SCP-1, SSX-2 and NY-ESO-1	ELISA	Breast cancer patients (100)	[69]
Thioredoxin-like 2 (TXNL2)	Protein array, dot blot	Discovery phase, breast cancer (<10)	[70]
interleukin 29, osteoprotegerin, survivin, growth hormone, and resistin	Autoantibody Profiling System (APS)	Discovery phase, breast cancer (<10)	[71]
CYP4Z1	ELISA	Breast cancer (19), controls (11)	[72]
p16, c-myc, TP53, and ANXA-1	ELISA	Breast cancer (102), controls (146)	[73]
Thymidylate synthase (TYMS) and C-terminal LIM domain protein 1 (PDLIM1)	ELISA	Breast cancer (30), controls (30)	[74]
Estrogen receptor alpha	ELISA	Breast cancer (48)	[75]
ALDOA, ENO1, GAPDH, PKM2, and TPI1	Proteomics, ELSIA	Prediagnostic ER+/PR+ breast cancer (48), healthy controls (65)	[76]
RBP-Jκ, HMGN1, PSRC1, CIRBP, and ECHDC1	ELISA	Invasive breast cancer (IBC, 59), ductal carcinoma in situ (DCIS, 61)	[77]
